# Changes in the Conformational State of Hemoglobin in Hemodialysed Patients with Chronic Renal Failure

**DOI:** 10.1155/2015/783073

**Published:** 2015-03-18

**Authors:** Anna Pieniazek, Krzysztof Gwozdzinski

**Affiliations:** ^1^Department of Thermobiology, University of Lodz, Pomorska 141/143, 90-236 Lodz, Poland; ^2^Department of Molecular Biophysics, University of Lodz, Pomorska 141/143, 90-236 Lodz, Poland

## Abstract

The aim of this study was to evaluate the properties of internal components of erythrocytes in chronic renal failure (CRF) patients undergoing hemodialysis (HD) in comparison to control subjects. For investigation of conformational state of hemoglobin and nonheme proteins (NHP) the maleimide spin label (MSL) in electron paramagnetic resonance (EPR) was applied. The studies were performed using MSL in whole cells and hemolysate as well as proteins separated by ion exchange chromatography and checked by electrophoresis. Additionally the level of –SH groups in hemolysate and isolated internal proteins of CRF erythrocytes was determined using 4,4′-dithiodipyridine. All measurements were performed before and after hemodialysis. Oxidative stress accompanying CRF/hemodialysed patients caused a significant decrease in the mobility of internal components inside erythrocytes indicated by MSL (*P* < 0.02). The significant decrease in mobility of spin labeled HbA_1c_ and HbA both before and after HD (*P* < 0.0002) as well as in nonheme proteins before hemodialysis (*P* < 0.05) versus control was indicated. Decrease in mobility of internal components of erythrocytes was accompanied by loss of thiols before and after hemodialysis versus control in NHP (*P* < 0.05), HbA_1c_ (*P* < 0.0002), and HbA (*P* < 0.0005). These findings showed oxidative influence of hemodialysis on hemoglobins and internal nonheme proteins in erythrocytes of CRF patients.

## 1. Introduction

Chronic renal failure is a debilitating condition which is responsible for high morbidity and mortality of suffering patients. The bad condition of the patient may be exacerbated due to the constant presence of uremic toxins and oxidative stress. In numerous studies it has been reported that reactive oxygen species (ROS) play a crucial role in the pathophysiological pathways of chronic renal failure [1, 2]. In CRF patients undergoing hemodialysis (HD) treatment the formation of reactive oxygen species is amplified, due to bioincompatible dialysis membrane. The oxidative stress is a result of polymorphonuclear leukocyte activation by the contact of blood with nonbiological materials, mainly dialysis membrane in the extracorporeal circuit during hemodialysis [3, 4].

The presence of ROS can cause damage in many molecules, such as lipids, proteins, carbohydrates, and DNA [5, 6]. Structural changes in albumin and increase in lipid peroxidation in plasma were observed in patients with chronic renal failure [7]. In patients undergoing hemodialysis an increase in advanced glycoxidation end products (AGEs), carbonyl groups, and advanced oxidation protein products (AOPP) was also observed [5, 8].

Oxidative stress in CRF patients can be additionally exacerbated by the depletion of antioxidative defenses in cells. The decrease in antioxidant enzyme activities as well as in low molecular weight antioxidants was found [9–11]. In these conditions, the main erythrocyte metabolic pathways are inefficient. For example, it results in decreased level of NADPH and GSH and increased activity of glutathione reductase (GSSG-R) [10, 12].

In patients with chronic renal failure numerous changes in the structure of the cell membrane of erythrocytes have been observed. The lipid composition and fatty acid content in CRF erythrocytes are disturbed [13, 14]. The higher content of unsaturated fatty acids leads to their greater propensity to be oxidized. Oxidative stress leads also to alteration in membrane cytoskeleton [15]. In our previous work, we showed changes in dynamics and conformation state of proteins in plasma membrane of erythrocytes from CRF patients. The RBC membrane was also more sensitive for oxidative stress than in healthy volunteers [14, 16]. In consequence, these processes further deteriorate physiological functions of erythrocytes or may even be the reason of cellular lysis. Structural changes in the cell membrane may result from the interaction of its components with uremic toxins and/or free radicals. Those processes may contribute to the alterations in the enzymatic activity of membrane bound proteins and may result in protein-protein as well as protein-lipid interactions. The activity of acetylcholinesterase (AChE) reflects changes in the chemical and physical properties of the hydrophobic environment of the membrane in the response to oxidative stress [17].

Furthermore, these changes in lipid composition and physical properties of the membrane may also result in erythrocyte aging process or premature removal from the circulation. Survival of the CRF patients cells is decreased because in patients treated with hemodialysis uremic erythrocytes are eliminated from the circulation earlier and a process of production of new cell occurs to compensate for early loss of old cells [18, 19]. Hence, the uremic blood is enriched with younger cells. Both high levels of uremic toxins and oxidative stress may have influence on higher incidence of complications such as anemia, atherosclerosis, cardiovascular disease, and accelerated aging processes [20].

In the present study, we have investigated the conformational state of pure hemoglobin HbA, HbA_1c_, and nonheme proteins of chronic renal failure patients before and after hemodialysis using electron paramagnetic resonance spin labeling method. The level of −SH groups in erythrocyte cytosol in CRF patients before and after hemodialysis was also examined.

Our findings provide evidence that CRF patients have impaired hemoglobin structure and these properties are enhanced during hemodialysis.

## 2. Materials and Methods

### 2.1. Chemicals

4-Maleimido-2,2,6,6,-tetramethylpiperidine-1-oxyl (MSL) and 4,4′-dithiodipyridine were obtained from Sigma Chemical Co. (St. Louis, MO). All other chemicals were analytical grade products from POCh (Gliwice, Poland).

### 2.2. Patients

The study population consisted of 10 patients, who had mild to advanced chronic renal failure (CRF) and who were treated at the Department of Internal Medicine at the Medical University in Lodz. Among them 8 patients were with glomerulonephritis, 1 with diabetic nephropathy, and 1 with polycystic kidney disease. Patients were dialyzed with the usage of polysulfone dialyzers LO PS 18 and dialysis fluid containing bicarbonate buffer for 3.5 to 4.5 hours, three times per week, as prescribed. Recruited patients (10 males) were between 39 and 74 years old, undergoing hemodialysis for 58 ± 11 months. All patients received erythropoietin.

Blood from patients was taken by their agreement and doctor acceptation and consent of the Bioethical Commission from the University of Lodz (KBBN-UL/4/2012). All subjects signed an informed consent form prior to participation.

The control group of 10 healthy men (45–61 years old) was recruited among volunteers of the Outpatient Center of Medical University in Lodz.

### 2.3. Preparation of Erythrocytes

Venous blood samples were collected (in standard sterile polystyrene vacuum tubes with heparin 16 U/mL of blood) before dialysis, in 20 minutes of dialysis session, in 1 hour of dialysis session, and immediately after dialysis, centrifuged, and washed three times with PBS. Packed cells were suspended in PBS (10 mM phosphate buffered saline, pH 7.4) to a hematocrit of 50%.

### 2.4. Spin Labeling of Erythrocytes

Erythrocytes were labeled with MSL by introduction in ethanol solution (0.1 mol/L) into the erythrocyte suspension and incubation for 1 hour at room temperature. The unbound label was removed by several washings with cold phosphate buffer pH 7.4 until an ESR signal in supernatant disappeared. From EPR spectra of spin labeled erythrocytes relative rotational correlation time (*τ*_*c*_) was calculated, according to the Kivelson formula [21] in modification of Keith and coworkers [22]:

(1) $$\begin{array}{c} \tau_{c}=kw_{0}\left(\sqrt{\frac{h_{0}}{h_{-1}}}-1\right), \end{array}$$

where *τ*_*c*_ is time when the spin label undergoes full rotation, *k* is constant equal to 6.5 × 10^−10^ s, *w*_0_ is width of the midline of spectrum, *h*_0_ is height of the midline of spectrum, and *h*_−1_ is height of the high-field line of spectrum.

### 2.5. Hemoglobin Preparation and Purification

Hemoglobin from washed red blood cells was prepared by Drabkin method, by the addition of water and toluene [23]. Hemolysate was centrifuged 4 000 ×g. After removing organic phase hemolysate was dialyzed against 20 mmol/L phosphate buffer pH 7.4 for 24 h. Hemoglobin (Hb) was purified using ion exchange chromatography according to Huisman et al. method [24] with modification on carboxymethylcellulose equilibrated with 20 mmol/L phosphate buffer pH 6.5 using a linear gradient of pH (6.5–8.0). The absorbance of the colorless fraction of the eluent was measured at 280 nm.

### 2.6. Polyacrylamide Gel Electrophoresis

SDS polyacrylamide gel electrophoresis was carried out in the presence of dithiothreitol on internal proteins separated by ion exchange chromatography. The fractions containing nonheme protein, HbA_1c_, and HbA were determined by electrophoresis in polyacrylamide gel in Tris-glycine buffer with SDS in the presence of dithiothreitol. Protein bands were stained with Coomassie blue R250.

### 2.7. Spin Labeling of Hemoglobin and Nonheme Proteins

To investigate conformational changes of hemoglobin and nonheme proteins maleimide spin label was applied. Crude hemoglobin as well as fractions of HbA_1c_, HbA, and nonheme proteins was labelled using (0.1 mol/L) ethanol solutions of MSL (50 : 1) and incubated for 2 hours at 4°C. Unbound spin label in all fractions was removed by 24 hours of dialysis against 10 mol/L phosphate buffer.

The mobility of the attached spin label was estimated by calculating the rotational correlation time (*τ*_*c*_) according to the Kivelson formula [21] with Keith et al. modification [22].

### 2.8. Thiol Measurements

The concentration of thiol groups in crude hemoglobin, HbA_1c_, HbA, and nonheme proteins was measured by the method of Egwim and Gruber [25]. Samples were diluted with 10 mmol/L phosphate buffer, pH 8.0, containing 1% of SDS and then 4,4′-dithiodipyridine (DTDP) from a 3 mmol/L stock solution was added and incubated for 1 hour at 37°C. Thiols react with 4,4′-dithiodipyridine to form 2-thiopyridone optically active at 324 nm. The basal optical activity of the samples was measured before the addition of 4,4′-dithiodipyridine. A calibration curve was prepared from different concentrations of reduced glutathione. The concentration of thiol groups was calculated as *μ*mol −SH/mg protein.

### 2.9. Protein Concentration

Protein concentration was evaluated using the Folin reagent by the spectrophotometric method according to Lowry et al. [26]. Hemoglobin concentration was measured using the Drabkin method [23].

### 2.10. EPR Measurement

EPR spectra were recorded at room temperature (21 ± 1°C) using a Bruker ESP 300 E X-band spectrometer, operating at a microwave frequency of 9.73 GHz. The instrumental settings were as follows: center field 3480 G; scan range 80 G; modulation frequency 100 kHz; modulation amplitude 1 G.

### 2.11. Statistical Analysis

All data were expressed as mean ± standard deviation. Normality of data was tested using the Shapiro-Wilk test and variance homogeneity was verified with Fisher test. The significance of the differences between couples of means was estimated using one-way ANOVA and post hoc Tukey test. The power of used test was checked for each analysis and always was more than 80%. The required sample size has been calculated before the data were pooled based on the STATISTICA.PL v.10. Statistical significance was accepted at *P* < 0.05.

## 3. Results

**Figure 1:**
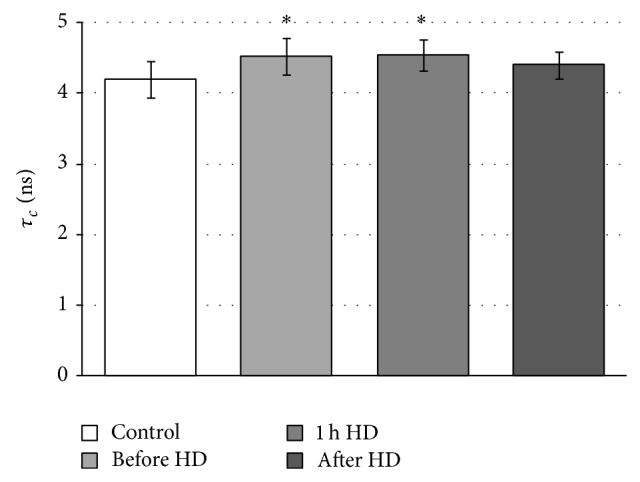
Changes in relative rotational correlation time of maleimide spin label inside erythrocytes from CRF patients before, in the first h of hemodialysis, and after hemodialysis. ^*^Significant difference, control versus before HD (*P* < 0.02) and control versus 1 h HD (*P* < 0.02).

The mobility of cytoplasmic peptides and proteins in whole RBCs was investigated by covalently bound maleimide spin label. MSL easily penetrates the erythrocyte membrane and can react with −SH groups of internal. It has been shown that more than 90% of bound spin label stays in the cytosol and it can be suspected that MSL binds inside of erythrocyte mainly with glutathione and much less with hemoglobin and membrane proteins [27]. However, its motion reflects changes in internal environment. From EPR spectra of MSL attached to glutathione inside erythrocytes relative rotational correlation time was determined.  Figure 1 shows the effect of hemodialysis on internal viscosity, which can reflect proteins and peptides mobility in whole erythrocytes before, in the first hour, and after hemodialysis.

Analysis of the maleimide spectrum shows significant increase in this parameter before and 1 hour of hemodialysis comparing with healthy donors. This parameter indicates a decrease in the segmental motion of peptides and proteins before and in the first hour of hemodialysis. This study provides evidence about changes in viscosity and other changes in the internal environment of the erythrocytes. After hemodialysis slight decrease in rotational correlation time comparing with sample before HD was observed.

**Figure 2:**
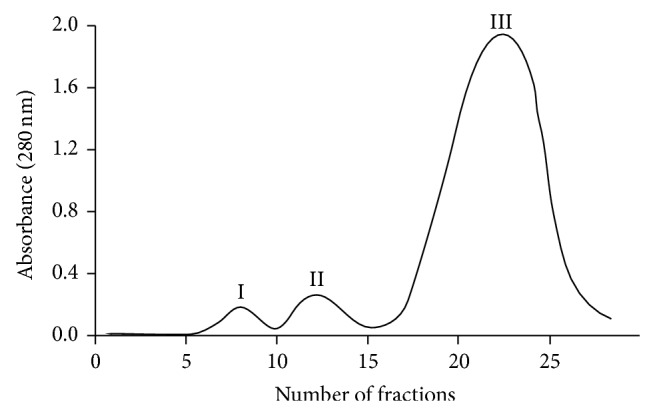
The chromatographic profile of the protein fractions obtained during hemolysate purification on carboxymethylcellulose.

Hemolysate proteins were separated by ion exchange chromatography on carboxymethylcellulose with a linear gradient of pH (Figure 2).

**Figure 3:**
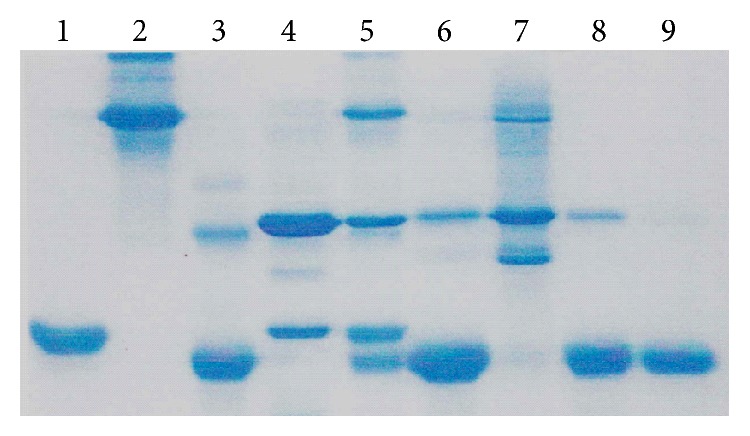
Electrophoresis results of SDS polyacrylamide gel in reduced conditions. Fractions of proteins obtained from hemolysate from patients with chronic renal failure undergoing hemodialysis after separation on carboxymethylcellulose. The markers were also used: (1) myoglobin; (2) bovine albumin; (3) cytochrome c; (4) anhydrase; (5) mixture of 1, 2, 3, and 4; (6) hemolysate (crude hemoglobin); (7) proteins in fraction I; (8) proteins in fraction II; and (9) fraction III (pure HbA). Protein bands were stained with Coomassie blue R250.

Fraction I contains nonheme proteins, fraction II HbA_1c_, and main fraction III pure hemoglobin (HbA). Band II marked as Hb_1c_ in fact contains a mixture of Hb_1c_ and Hb_1_, as well as carbamylated hemoglobin. Since diabetes mellitus can lead to chronic renal failure many CRF patients possess glycated and carbamylated haemoglobin. These fractions were analysed using gel electrophoresis (Figure 3).

Separated fractions: nonheme proteins I, hemoglobin HbA_1c_ II, and pure HbA III were examined using EPR. The alterations in hemolysate (crude hemoglobin) after cell lysis as well as in fractions I, II, and III were detected using maleimide spin label.

**Figure 4:**
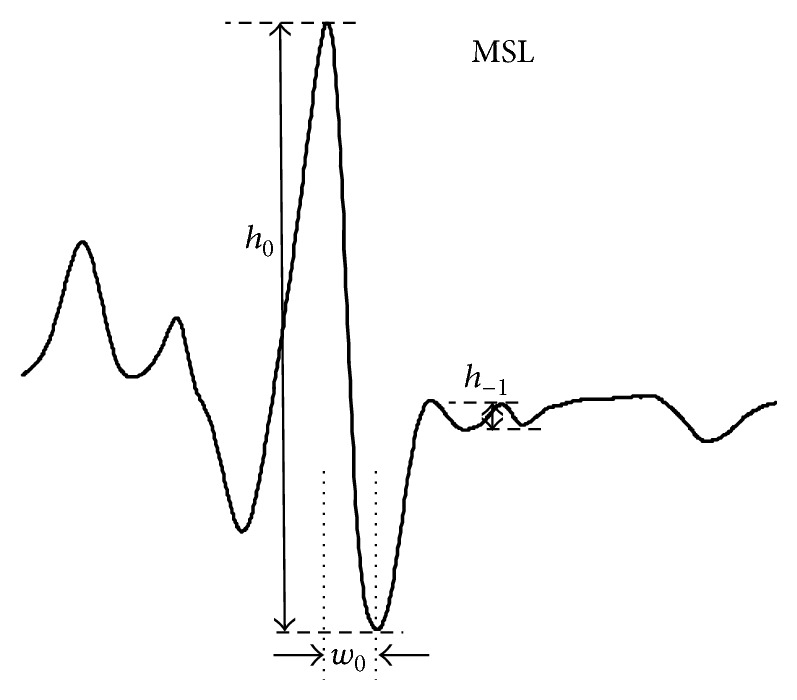
EPR spectrum of MSL attached to hemoglobin (HbA).

In pH 7–7.5 MSL reacts with −SH groups of proteins [28]. It has been reported that MSL attaches to *β*Cys93 in hemoglobin [29]. The high reactivity of Cys-93(*β*) of HbA with maleimide in oxy-hemoglobin conformation is the basis for the HbA assay.  Figure 4 shows maleimide (d) attached to purified HbA.

**Figure 5:**
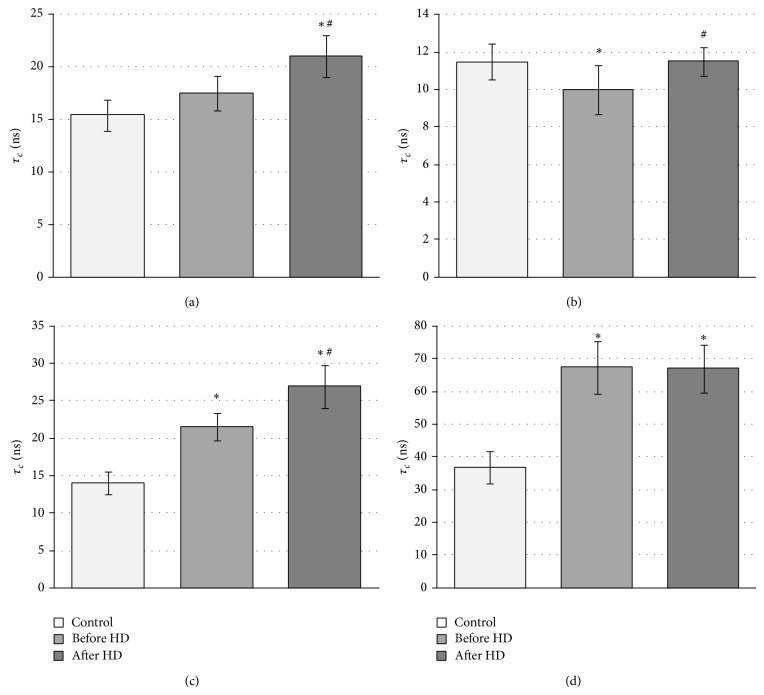
Changes in rotational correlation time of MSL attached to erythrocytes cytoplasmic proteins from patients with chronic renal failure undergoing hemodialysis (HD). (a) Crude hemoglobin (^*^significant difference, control versus after HD (*P* < 0.0002); ^#^significant difference before HD versus after HD (*P* < 0.002)); (b) nonheme proteins (^*^significant difference control versus before HD (*P* < 0.05); ^#^significant difference before HD versus after HD (*P* < 0.05)); (c) hemoglobin A_1c_ (^*^significant difference, control versus before HD and after HD (*P* < 0.0002); ^#^significant difference before HD versus after HD (*P* < 0.0005)); (d) hemoglobin A (^*^significant difference, control versus before HD and after HD (*P* < 0.0002)).

This spectrum consists of strongly immobilized fractions of the label. From EPR spectra the relative rotational correlation time was calculated.  Figure 5 shows changes in relative rotational correlation times of MSL attached to hemolysate, fraction I, and hemoglobins: A_1c_ and A. Generally the significant increase of *τ*_*c*_ before and after hemodialysis in comparison to control was found. We observed the increase in this parameter in hemolysate (Figure 5(a)) and hemoglobin fractions (Figures 5(c) and 5(d)) of CRF patients before and after hemodialysis. The significant decrease in the mobility of spin labeled hemoglobin A_1c_ and HbA both before and HD was observed at *P* < 0.0002 as well as crude hemoglobin (hemolysate) after hemodialysis was also noted. In the case of nonheme proteins the significant decrease in *τ*_*c*_ before hemodialysis (*P* < 0.05) was found. Generally, the obtained results showed also a significant decrease in the motion of spin labeled erythrocyte components after hemodialysis in comparison to before HD.

**Figure 6:**
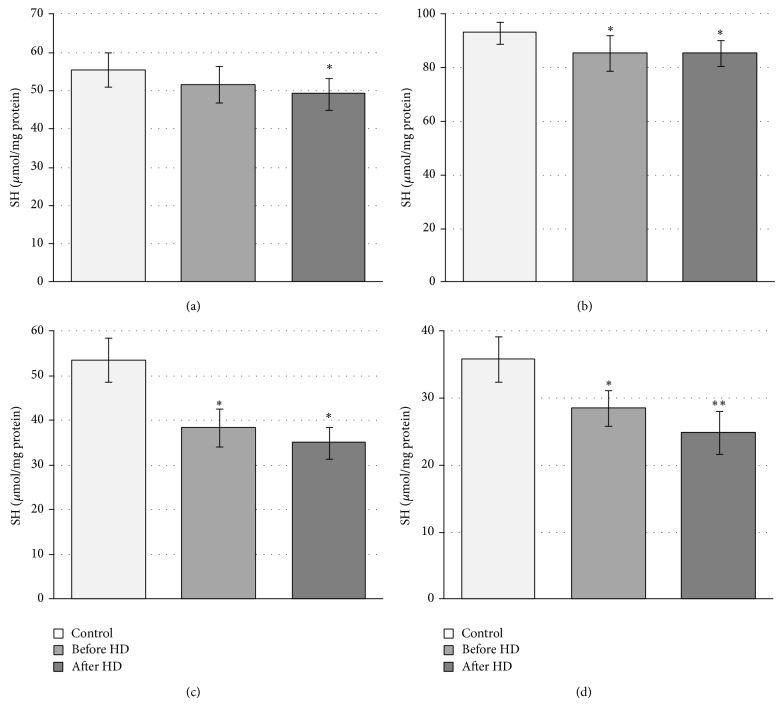
Changes in free thiol groups concentration of cytoplasmic proteins from patients with chronic renal failure undergoing hemodialysis (HD). (a) Crude hemolysate (^*^significant difference, control versus After HD (*P* < 0.05)); (b) nonheme proteins (^*^significant difference control versus before HD and after HD (*P* < 0.05)); (c) hemoglobin A_1c_ (^*^significant difference, control versus before HD and after HD (*P* < 0.0002)); (d) hemoglobin A (^*^significant difference, control versus before HD (*P* < 0.0005); ^*^significant difference, control versus after HD (*P* < 0.0002)).

The level of thiol groups in all fractions was studied. The significant decline in −SH groups in hemolysate after HD as well as in all other fractions both before and after hemodialysis was found (Figure 6).

## 4. Discussion

In chronic renal failure, besides loss of endocrine function also loss of excretory function occurs, which leads to the accumulation of uremic toxins in the blood. These compounds may influence on properties of red blood cells, including their metabolism, shape, deformability, and in consequence life span [18, 19].

Erythrocytes and plasma components in CRF patients can be damaged by higher concentrations of urea in the blood, which can lead to carbamylation of lipids, proteins, and nucleic acids [30]. Another cause of uremic RBC damage is the action of mechanical forces during hemodialysis generated by rotary blood pumps [31, 32].

In HD patients, direct contact of blood cells with dialysis membrane leads to activation of neutrophils, monocytes or platelets, accumulation of oxidized components and prooxidant compounds in the blood, and depletion of low molecular weight antioxidants. Activation of phagocytic cells leads to respiratory burst and the trigger in reactive oxygen species production, mainly superoxide anion radical and hydrogen peroxide, which was shown by fluorescent probes [3, 33, 34]. The formation of the most reactive hydroxyl radical during hemodialysis was showed directly by the spin trapping method in spectroscopy of electron paramagnetic resonance [35, 36]. Oxidative stress can be also induced by ferryl and a radical ferryl form of hemoglobin and methemoglobin, which are released from disrupted RBCs [37]. ROS induces damage of biological material in the blood and cell injury including erythrocytes. Oxidative stress generated during hemodialysis is the most important source of cells injury and plasma component damage. ROS production from external and internal sources is accompanied with lipid and protein oxidation in plasma and erythrocyte membrane.

Since the cell membrane of erythrocytes is the first target of action of ROS many works were devoted to the study of its properties. Therefore, the aim of this study was the investigation of the influence of hemodialysis on erythrocyte internal components, mainly hemoglobin because this hemoprotein is 95% of all proteins inside the erythrocyte. Application of EPR spectroscopy in conjunction with spin labeling technique allowed us to examine internal component changes and to determine alterations in the conformational state of proteins including hemoglobin. Moreover, spin labels through binding to macromolecules can act as reporting groups, providing information about changes in their microenvironment.

The alterations in components inside the cells were studied by maleimide spin label, which can penetrate erythrocyte membrane and can react with −SH groups of peptides and proteins but mainly with glutathione [27, 38]. The significant (*P* < 0.02) increase in relative rotational correlation time which reflects decrease in the motion was found before and in the 60th min. of hemodialysis in comparison to healthy donors. However, a slight decrease in this parameter after hemodialysis comparing with the sample before HD was observed, indicating partially reversible changes in internal fluid. The observed changes can be the result of internal components oxidation which can lead to their conformational changes or/and hemoglobin binding to the membrane as well as changes in RBC volume. It has been shown that binding of hemoglobin to membrane is correlated with the level of oxidative stress and may be used as a marker of oxidative damage [39].

The alterations in the internal component mobility in erythrocytes were the reason for their more detailed studies. The erythrocytes were lysed and hemolysate was chromatographed using ion exchange chromatography. Three main fractions were analyzed using polyacrylamide gel electrophoresis. Hemolysate and three fractions containing a mixture of nonheme proteins, hemoglobin A_1c_, and HbA, respectively, were labeled with MSL. Using maleimide spin label we observed a statistically significant increase in rotational correlation time in hemolysate (crude hemoglobin) after hemodialysis (*P* < 0.0002) in comparison to control. However, insignificant increase of *τ*_*c*_ before hemodialysis was also noted. This parameter was also applied for estimation of changes in proteins in fractions I, II, and III. It has been shown that maleimide spin label attaches to −SH groups in cysteine-93 of *β*-globin chains of Hb [40, 41]. However, the spectrum of spin-labeled hemoglobin has two different spatial orientations relative to the hemoglobin molecule and exhibited considerable differences in local anisotropic motion due to the binding site of Hb [40, 41]. The significant increase in rotational correlation time of MSL attached to globin chains before and after hemodialysis was observed for hemoglobin HbA_1c_ and HbA. In HbA_1c_ significant difference (Figure 5(c)) in this parameter before and after HD was found. In the case of HbA of CRF patients it was approximately 2-fold higher than in control and did not change after HD. On the other hand, decrease in rotational correlation time before dialysis in the fraction which contains a mixture of nonheme proteins was found, but after HD *τ*_*c*_ increased to the control value.

Differences in rotational correlation time of MSL attached to hemoglobin may be a result of conformational changes in the local environment of globin chain in the binding site of labels.

In the case of HbA of CRF patients, the decrease of rotational correlation time was observed. The changes in conformation of globin chain caused an increase in freedom of molecular motion of label. Our finding is in agreement with results of Ogawa and McConnell who showed that oxygenation of haemoglobin leads to significant structural changes near the reactive −SH group in *β*-chain [29].

The increase in rotational correlation times in spin labeled hemoglobins and nonheme proteins for MSL before hemodialysis may be a result of oxidation. In the previous paper, it has been shown that carbamylated membrane proteins are more sensitive for oxidative stress than native [14]. However, the obtained results showed also that hemodialysis leads to higher changes in the conformational state of hemoglobin and other proteins, which is a consequence of oxidative stress occurring during dialysis. It has been shown that during hydrogen peroxide hemoglobin oxidation, *β*Cys93 and *β*Cys112 residues are irreversibly oxidized to cysteic acid [42]. Both cysteine oxidation and oxidative modifications of tryptophan and methionine lead to loss of *α*-helical structure of the *β*-chain of globin [42].

Because the thiol groups of cysteines in hemoglobin can be easily oxidized during oxidative stress, their level in all fractions during hemodialysis was studied. The significant decrease in the level of −SH groups in hemolysate after HD as well as in all other fractions both before and after hemodialysis was observed. Moreover, the level of thiols was significantly lower after hemodialysis in comparison to before HD. These findings confirm the results obtained for both hemoglobins and nonheme proteins and are linked also to oxidative stress, which occurs during hemodialysis. The high valence of iron in heme initiates oxidative reactions with similar reactivity to hydroxyl radical in vivo [43, 44]. In these conditions −SH groups in proteins are oxidized to sulfenic, sulfinic, or sulfonic acid residues or/and associated with the promotion of both fragmentation and aggregation of proteins.

It is possible that the alterations in the structure of hemoglobins and other proteins as well as thiols level after hemodialysis inside erythrocytes are results of oxidation by internal reactive oxygen species generated during dialysis.

Autoxidation of hemoglobin produces methemoglobin and superoxide anion radical. In healthy donors, 3% of total hemoglobin is converted to MetHb daily. Superoxide is dismutated by CuZnSOD to hydrogen peroxide, which can react with hemoglobin and methemoglobin giving strongly oxidizing agents ferryl and radical ferryl forms. However, in normal cells, a balance between ROS formation and their detoxification exists and oxidized hemoglobin is regenerated to functional reduced form by metHb reductase dependent on NADH [45]. In CRF erythrocytes, this equilibrium is disturbed because they have impaired antioxidant defense systems. The decrease in the activity of SOD, decrease in the level of GSH, and higher GSSG/GSH ratio were described in RBC from hemodialysed patients [8, 46, 47].

It has been shown that both hemoproteins Hb and Mb in oxidized state, for example, in ferryl and ferryl radical forms, can induce lipid peroxidation by abstraction of a hydrogen atom in the hydrocarbon chain [48]. These hemoproteins are responsible for oxidative injury associated with rhabdomyolysis, hemolysis of erythrocytes, and brain hemorrhage [49]. We suggest that hemoglobin and other nonheme proteins can be damaged by ROS generated inside erythrocytes. It is also possible that structurally modified hemoglobin in patients with CRF has greater catalytic possibilities in generation of ROS than normal hemoprotein. It has been reported that impaired hemoglobin in sickle cell anemia increases oxidative stress in red blood cells [50].

On the other hand, internal components of erythrocytes can be also damaged by ROS from external sources, for example, released from phagocytic cells including hypochlorous acid and nitric oxide during respiratory burst [51–53]. Nitric oxide can easily penetrate plasma membrane and its reaction with oxygen and superoxide anion generates nitrogen dioxide and peroxynitrite, respectively, which are powerful oxidizing and nitrating agents in vivo [54, 55]. Enhanced production of nitric oxide and tyrosine nitration in plasma of hemodialysed patients was found [53, 55].

## 5. Conclusions

In this paper, we showed that hemodialysis of CRF patients enhanced oxidative damage of erythrocyte components. HD led to decrease in the mobility of spin labeled hemoglobin and nonheme proteins. Hemodialysis sessions led also to a loss of thiols in Hb and nonheme proteins.

## References

[1] Hambali Z., Ahmad Z., Arab S., Khazaai H. (2011). Oxidative stress and its association with cardiovascular disease in chronic renal failure patients. *Indian Journal of Nephrology*.

[2] Popolo A., Autore G., Pinto A., Marzocco S. (2013). Oxidative stress in patients with cardiovascular disease and chronic renal failure. *Free Radical Research*.

[3] Himmelfarb J., Lazarus J. M., Hakim R. (1991). Reactive oxygen species production by monocytes and polymorphonuclear leukocytes during dialysis. *American Journal of Kidney Diseases*.

[4] Ekdahl K. N., Lambris J. D., Elwing H. (2011). Innate immunity activation on biomaterial surfaces: a mechanistic model and coping strategies. *Advanced Drug Delivery Reviews*.

[5] Antolini F., Valente F., Ricciardi D., Baroni M., Fagugli R. M. (2005). Principal component analysis of some oxidative stress parameters and their relationships in hemodialytic and transplanted patients. *Clinica Chimica Acta*.

[6] Valentini J., Grotto D., Paniz C., Roehrs M., Burg G., Garcia S. C. (2008). The influence of the hemodialysis treatment time under oxidative stress biomarkers in chronic renal failure patients. *Biomedicine and Pharmacotherapy*.

[7] Pieniazek A., Brzeszczynska J., Kruszynska I., Gwozdzinski K. (2009). Investigation of albumin properties in patients with chronic renal failure. *Free Radical Research*.

[8] Nguyen-Khoa T., Massy Z. A., Pascal De Bandt J. (2001). Oxidative stress and haemodialysis: role of inflammation and duration of dialysis treatment. *Nephrology Dialysis Transplantation*.

[9] Cao G., Prior R. L. (1998). Measurement of oxygen radical absorbance capacity in biological samples. *Methods in Enzymology*.

[10] Rico M. G., Puchades M. J., Ramón R. G., Sáez G., Tormos M. C., Miguel A. (2006). Effect of oxidative stress in patients with chronic renal failure. *Nefrologia*.

[11] Libetta C., Sepe V., Esposito P., Galli F., Dal Canton A. (2011). Oxidative stress and inflammation: implications in uremia and hemodialysis. *Clinical Biochemistry*.

[12] Stepniewska J., Dolegowska B., Ciechanowski K., Kwiatkowska E., Millo B., Chlubek D. (2006). Erythrocyte antioxidant defense system in patients with chronic renal failure according to the hemodialysis conditions. *Archives of Medical Research*.

[13] Witkowska J., Bober J., Chlubek D. (2007). Changes in the lipid content of erythrocytes in patients with chronic renal failure. *Annales Academiae Medicae Stetinensis*.

[14] Brzeszczynska J., Luciak M., Gwozdzinski K. (2008). Alterations of erythrocyte structure and cellular susceptibility in patients with chronic renal failure: effect of haemodialysis and oxidative stress. *Free Radical Research*.

[15] Olszewska M., Wiatrow J., Bober J. (2012). Oxidative stress modulates the organization of erythrocyte membrane cytoskeleton. *Postepy Higieny i Medycyny Doswiadczalnej*.

[16] Stoya G., Klemm A., Baumann E. (2002). Determination of autofluorescence of red blood cells (RBCs) in uremic patients as a marker of oxidative damage. *Clinical Nephrology*.

[17] Srivastava N., Sharma R. K., Singh N., Sharma B. (2012). Acetylcholinesterase from human erythrocytes membrane: a screen for evaluating the activity of some traditional plant extracts. *Cellular and Molecular Biology*.

[18] Costa E., Rocha S., Rocha-Pereira P. (2008). Changes in red blood cells membrane protein composition during hemodialysis procedure. *Renal Failure*.

[19] Antonelou M. H., Kriebardis A. G., Velentzas A. D., Kokkalis A. C., Georgakopoulou S.-C., Papassideri I. S. (2011). Oxidative stress-associated shape transformation and membrane proteome remodeling in erythrocytes of end stage renal disease patients on hemodialysis. *Journal of Proteomics*.

[20] Bhogade R. B., Suryakar A. N., Joshi N. G., Patil R. Y. (2008). Effect of vitamin E supplementation on oxidative stress in hemodialysis patients. *Indian Journal of Clinical Biochemistry*.

[21] Kivelson D. (1960). Theory of ESR linewidths of free radicals. *The Journal of Chemical Physics*.

[22] Keith A., Bulfield G., Snipes W. (1970). Spin-labeled neurospora mitochondria. *Biophysical Journal*.

[23] Drabkin D. L. (1946). Spectrophotometric studies; the crystallographic and optical properties of the hemoglobin of man in comparison with those of other species. *The Journal of Biological Chemistry*.

[24] Huisman T. H. J., Martis E. A., Dozy A. (1958). Chromatography of hemoglobin types on carboxymethylcellulose. *The Journal of Laboratory and Clinical Medicine*.

[25] Egwim I. O. C., Gruber H. J. (2001). Spectrophotometric measurement of mercaptans with 4,4′-dithiodipyridine. *Analytical Biochemistry*.

[26] Lowry O. H., Rosebrough N. J., Farr A. L., Randall R. J. (1951). Protein measurement with the Folin phenol reagent. *The Journal of Biological Chemistry*.

[27] Gwozdzinski K. (1991). A spin label study of the action of cupric and mercuric ions on human red blood cells. *Toxicology*.

[28] Berliner L. J. (1983). The spin-label approach to labeling membrane protein sulfhydryl groups. *Annals of the New York Academy of Sciences*.

[29] Ogawa S., McConnell H. M. (1967). Spin-label study of hemoglobin conformations in solution. *Proceedings of the National Academy of Sciences of the United States of America*.

[30] Jaisson S., Pietrement C., Gillery P. (2011). Carbamylation-derived products: bioactive compounds and potential biomarkers in chronic renal failure and atherosclerosis. *Clinical Chemistry*.

[31] Sakota D., Sakamoto R., Sobajima H. (2008). Mechanical damage of red blood cells by rotary blood pumps: selective destruction of aged red blood cells and subhemolytic trauma. *Artificial Organs*.

[32] Polaschegg H.-D. (2009). Red blood cell damage from extracorporeal circulation in hemodialysis. *Seminars in Dialysis*.

[33] Cristol J. P., Canaud B., Rabesandratana H., Gaillard I., Serre A., Mion C. (1994). Enhancement of reactive oxygen species production and cell surface markers expression due to haemodialysis. *Nephrology Dialysis Transplantation*.

[34] Bonomini M., Stuard S., Carreno M. P. (1997). Neutrophil reactive oxygen species production during hemodialysis: role of activated platelet adhesion to neutrophils through P-selectin. *Nephron*.

[35] Gwozdzinski K., Janicka M. (1995). Oxygen free radicals and red blood cell damage in acute renal failure. *Biochemical Society Transactions*.

[36] Gwoździński K., Janicka M., Brzeszczyńska J., Luciak M. (1997). Changes in red blood cell membrane structure in patients with chronic renal failure. *Acta Biochimica Polonica*.

[37] Jeney V., Eaton J. W., Balla G., Balla J. (2013). Natural history of the bruise: formation, elimination, and biological effects of oxidized hemoglobin. *Oxidative Medicine and Cellular Longevity*.

[38] Daveloose D., Fabre G., Berleur F., Testylier G., Leterrier F. (1983). A new spin label method for the measurement of erythrocyte internal microviscosity. *Biochimica et Biophysica Acta*.

[39] Sharma R., Premachandra B. R. (1991). Membrane-bound hemoglobin as a marker of oxidative injury in adult and neonatal red blood cells. *Biochemical Medicine and Metabolic Biology*.

[40] Ohnishi S., Boeyens J. C., McConnell H. M. (1966). Spin-labeled hemoglobin crystals. *Proceedings of the National Academy of Sciences of the United States of America*.

[41] Boeyens J. C., McConnell H. M. (1966). Spin-labeled hemoglobin. *Proceedings of the National Academy of Sciences of the United States of America*.

[42] Jia P. W., Buehler R. A., Boykins R. M., Venable R. M., Alayash I. (2007). Structural basis of peroxide mediated changes in human hemoglobin: a novel oxidative pathwa. *The Journal of Biological Chemistry*.

[43] Harel S., Kanner J. (1988). The generation of ferryl or hydroxyl radicals during interaction of haemproteins with hydrogen peroxide. *Free Radical Research Communications*.

[44] Vollaard N. B. J., Reeder B. J., Shearman J. P., Menu P., Wilson M. T., Cooper C. E. (2005). A new sensitive assay reveals that hemoglobin is oxidatively modified in vivo. *Free Radical Biology and Medicine*.

[45] Xu F., Quandt K. S., Hultquist D. E. (1992). Characterization of NADPH-dependent methemoglobin reductase as a heme-binding protein present in erythrocytes and liver. *Proceedings of the National Academy of Sciences of the United States of America*.

[46] Shainkin-Kestenbaum R., Caruso C., Berlyne G. M. (1990). Reduced superoxide dismutase activity in erythrocytes of dialysis patients: a possible factor in the etiology of uremic anemia. *Nephron*.

[47] Knap B., Prezelj M., Buturović-Ponikvar J., Ponikvar R., Bren A. F. (2009). Antioxidant enzymes show adaptation to oxidative stress in athletes and increased stress in hemodialysis patients. *Therapeutic Apheresis and Dialysis*.

[48] Reeder B. J., Wilson M. T. (2005). Hemoglobin and myoglobin associated oxidative stress: from molecular mechanisms to disease states. *Current Medicinal Chemistry*.

[49] Reeder B. J., Sharpe M. A., Kay A. D., Kerr M., Moore K., Wilson M. T. (2002). Toxicity of myoglobin and haemoglobin: oxidative stress in patients with rhabdomyolysis and subarachnoid haemorrhage. *Biochemical Society Transactions*.

[50] Goswami K., Ray D. (2011). Putative pathogenic effect of oxidative stress in sickle cell disorder. *Biomedical Research*.

[51] Himmelfarb J., McMonagle E., McMenamin E. (2000). Plasma protein thiol oxidation and carbonyl formation in chronic renal failure. *Kidney International*.

[52] Sutherland W. H. F., de Jong S. A., Walker R. J. (2004). Hypochlorous acid and low serum paraoxonase activity in haemodialysis patients: an in vitro study. *Nephrology Dialysis Transplantation*.

[53] Amore A., Bonaudo R., Ghigo D. (1995). Enhanced production of nitric oxide by blood-dialysis membrane interaction. *Journal of the American Society of Nephrology*.

[54] Radi R., Peluffo G., Alvarez M. N., Naviliat M., Cayota A. (2001). Unraveling peroxynitrite formation in biological systems. *Free Radical Biology and Medicine*.

[55] Mitrogianni Z., Barbouti A., Galaris D., Siamopoulos K. C. (2004). Tyrosine nitration in plasma proteins from patients undergoing hemodialysis. *American Journal of Kidney Diseases*.

